# The Effects of Single-Bout Exercise Interventions with Different Exercise Modalities on Executive Function in Youths

**DOI:** 10.3390/sports12040086

**Published:** 2024-03-22

**Authors:** Chung-Kit Tam, Yu-Hua Chang, Kok-Hwa Tan

**Affiliations:** 1 Department of Kinesiology, National Tsing Hua University, Hsinchu City 300035, Taiwan; tamchungkit236@gapp.nthu.edu.tw; 2 Physical Education Office, National Tsing Hua University, Hsinchu City 300044, Taiwan; yhchang@mx.nthu.edu.tw

**Keywords:** closed-skill exercise, mixed-skill exercise, open-skill exercise, task switching

## Abstract

Background: This study aimed to investigate how single-bout open-skill exercise (OSE), closed-skill exercise (CSE), and mixed-skill exercise intervention (MSE) influence executive function. Method: A total of 120 students aged between 18 and 25 were separated into three groups: closed-skill exercise, open-skill exercise, and mixed-skill exercise. A task-switching test was performed before and after a single bout of exercise intervention. The simple reaction time, choice reaction time, switch cost, and correction rate were tested in a task-switching test. The results were analyzed via a two-way analysis of variance, with a significance level of α = 0.05, to compare the effects of the intervention. Results: Only open-skill exercise exhibited a significant effect on the simple reaction time (*p* < 0.05). In terms of choice reaction time and switch cost, all three intervention groups exhibited significant improvements, with no significant differences observed between the three groups (*p* < 0.05). The correction rate did not show a significant effect post-intervention, and no significant differences were observed between the groups. The correction rate showed no significant effect after the intervention or between groups. Conclusion: All three types of exercise can shorten choice reaction time and switch cost, but only OSE can reduce simple reaction time.

## 1. Introduction

Cognitive function is an important topic that encompasses various mental processes in our daily life, such as memory, computation, and executive function [1,2]. Executive function is one of the major ways in which sports improve cognitive function [3,4]. It includes several of the following components: inhibition, working memory, reaction time, and cognitive flexibility [1,5]. The reaction time indicates how quickly people can respond to stimuli [6]. There are three types of reaction time: simple reaction time, reaction time for recognition, and choice reaction time. Simple reaction time refers to responding to any stimulus. Reaction time for recognition entails a cognitive process whereby the most appropriate response is selected depending on the stimuli. Choice reaction time indicates that an individual must accurately respond to a specified stimulus when presented with multiple stimuli [7,8]. The reaction time increases with task switching, which is called the ‘switch cost’ [9,10].

According to the stage model of task switching proposed by Rubinstein et al. [8], task switching can be delineated into executive control processes and task processes. Executive control processes involve goal shifting and rule activation, and task processes include stimulus identification, response selection, and movement production. Various stimuli from visual or auditory sources activate distinct areas in the human brain [9]. Consequently, when the rules of a task-switching test are more intricate, longer reaction times are necessary as the brain requires additional time to process the received information.

For executive control processes, the stimulus and cue stored in the working memory and frontal cortex are responsible for the whole process; therefore, task switching has a strong relationship with our cognitive function [11].

Task switching is a common mental process involving the transfer of attention between different tasks or missions [12,13]. For example, when we take notes while on the phone, we engage in different types of task-switching. In the early twentieth century, Jersild [9] used both single and combined tasks to investigate people’s mental state and ability to task switch. In the single-task section, participants were asked to continuously add six or subtract three from a number. The sequence of the single-task section was presented as AAA or BBB. In the combined-task section, participants had to add six to the first number, subtract three from the second number, and so on. The sequence could be ABAB, it could alternate, or it could be more complex. In addition to fixed sequence alternations, random sequences and responses to certain targets are also commonly used in task-switching tests [14].

In recent years, there has been an increasing amount of research focusing on how exercise can influence the cognitive function. According to a study by Diamond [15], physical activities can help improve our executive function, and both a single bout of exercise and long-term exercise intervention have different effects. Single-bout exercise can help increase hemodynamic activity and neurotransmitter levels in our brain, which may help to improve attention, working memory, problem-solving abilities, cognitive flexibility, and verbal fluency. However, these benefits may vary based on the exercise intensity, duration, health condition, and specific cognitive performance assessment employed [16,17,18,19,20,21]. When long-term interventions are employed, exercise has been shown to contribute to improved cerebral blood flow, angiogenesis, neuroplasticity, brain-derived neurotrophic factor (BDNF) levels, and synaptic plasticity [17,22,23,24]. This corresponds to enhancements in visual–spatial perception, working memory, attention, reaction time, cognitive flexibility, processing speed, executive function, and conflict resolution [25,26,27,28].

Different types of single-bout exercises may provide different benefits to cognitive functions [29]. In McSween et al.’s [30] study, aerobic exercise intervention was found to improve cognitive performance in the elderly. This result is consistent across different age groups. For example, Nanda, Balde, and Manjunatha [21] performed a cognitive test on 10 healthy university students, comparing the results before and after 30 min of cycling at 60–70% HRR. The results showed that 30 min of moderate exercise can immediately improve memory, reasoning, and planning. However, a different result was reported in a study by Gothe et al. [31]. In the study, 30 female university students participated in a single bout of yoga or aerobic intervention, and a better performance in a flanker and N-back test was achieved after yoga intervention compared with aerobic intervention and baseline results. Additionally, moderate-to-high-intensity exercise has also been shown to help improve children’s cognitive functions [32]. In Williams et al.’s [33] review, exercise for 10 to 30 min was found to be most beneficial to children’s cognitive function and the benefits to cognitive function after exercise can last for 45 min. In a study by Chang et al. [34], 30 late-middle-aged adults participated in a single-bout resistance exercise and performed Stroop tests before and after the intervention. The result showed that a single bout of resistance training can also improve cognitive functions, particularly executive control.

As for exercise intensity, previous studies showed that acute moderate- or high-intensity exercise can improve working memory, long-term memory, and cognitive performance by improving encoding ability, retrieval function, and reticular system activation [35,36,37,38].

Additionally, different exercise modalities can also have distinct effects on cognitive functions. Exercises can be categorized as open-skill and closed-skill exercises [39]. Open-skill exercises, such as table tennis and basketball, refer to the exercise environment changes according to tactics and competitors, while closed-skill exercises, such as running, or cycling, are performed in a stable and predictable environment. Participants’ motions during closed-skill exercise are based on what they have practiced, whereas the motions performed during open-skill exercise change based on what happens during the competition [40]. Tsai et al. [41] investigated the effects of a 24-week open-skill exercise (table tennis) and closed-skill exercise (running or cycling) intervention compared with a control group (non-exercise). The results showed significant effects in both groups on reaction time and short-term memory in a task-switching test. In a systematic review by Zhu et al. [42], OSE exhibited a superior effect on cognitive performance compared with CSE, as analyzed in many cross-sectional studies. Moreover, a recent study reported that participants exhibited better performance in retrospective memory tasks after engaging in CSE, as compared with OSE [43]. Furthermore, Diamond [44] suggested that aerobic exercises that focus on a single element (such as running or spin bikes) may not effectively improve cognitive function; the effectiveness depends on participants’ emotions, age, and other various factors. Exercises that involve thinking, communication, or visual coordination (such as martial arts, Zumba, and football) are more effective in improving cognitive function.

Moreover, Müller et al. [45] suggested that age is also a reasonable factor influencing the cognitive benefits derived from exercise. However, a significant number of studies have predominantly focused on children, teenagers, and the elderly [41,46,47,48,49]. Previous research has also demonstrated that physical activities can improve cognitive performance and academic outcomes [50]. Since neural system development and the initial cognitive performance of youths are relatively mature, the cognitive effects on this population remain unclear and require further study [51].

Overall, it has been demonstrated that exercise has the potential to enhance executive function, reaction time, and working memory, and to prevent cognitive function deficits. However, most previous studies have focused on closed-skill exercises, with limited exploration into different exercise modalities. Additionally, due to the mature brain function in youth, it is challenging to discern significant effects when comparing against studies involving the elderly and children. Notably, few studies have investigated the impact of different exercise modalities on youths. Therefore, the focus of this study concerns understanding the acute effects of single-bout open-skill and closed-skill exercises and how a combination of the two may affect executive function. The second aim of this study concerns investigating whether exercise intervention has an impact on the cognitive development of youths.

## 2. Materials and Methods

### 2.1. Subjects

This study involved 120 university students, aged between 18 and 25 years old, without injuries that could affect exercise performance and with no history of brain, cognition, mental, or cardiovascular diseases. Moreover, the study excluded those with color vision deficiencies, and each participant was provided with a Physical Activity Readiness Questionnaire (PAR-Q) and an informed consent form which informed them of the purpose, procedures, and precautions relevant to the study. After signing the consent form, the participants provided basic information so that they could become subjects of this study. Recruitment was contingent upon the absence of any psychological or physical impediments.

### 2.2. Experimental Design

This experiment adhered to the protocol of Yu et al. [52]. Participants were grouped into three categories in accordance with a random sequence: ‘open-skill exercise group’ (OSE) (n = 40), ‘close-skill exercise group’ (CSE) (n = 40), and ‘mixed-skill exercise group’ (MSE) (n = 40). Each group consisted of 40 participants. Each participant underwent a pre-test of the task-switching test. After a 5 min break, a 35 min exercise intervention (including warm-up) was conducted by the researchers. To understand the acute effect of three exercise modalities, a post-test was administered within 5 min after the intervention was completed. Participants were prohibited from consuming caffeine 2 h before the experiment. Additionally, they were instructed to abstain from alcohol for 24 h before the experiments, and high-intensity exercise was not allowed immediately prior to the experiments.

#### 2.2.1. Pre-Test and Post-Test Protocol

The task-switching test was adapted from the cognitive switching tasks proposed by Yu, Chan, Chau, and Fu [52] and Kattner et al. [53], with some modifications. The test program was performed using PsychoPy (v2022.2.4) [54]. The test included a single-task test and a combined-task test, each consisting of 24 trials, examining participants’ simple reaction times and choice reaction times, respectively. A one-minute break was given between the two tests. All key presses during the experiment were required to be performed using the dominant hand. The distance between the participants and the monitor was determined by the participants. Before each test, the operational procedures were explained, and the participants had to confirm that they understood by pressing the ‘space’ bar. Eight example trials were conducted before the twenty-four formal trials, and the results of these example trials were excluded from statistical analysis. Following the example trials, a ‘Start’ signal appeared on the monitor, and it remained there for 1500 ms before the formal commencement of the experiment.

**Figure 1:**
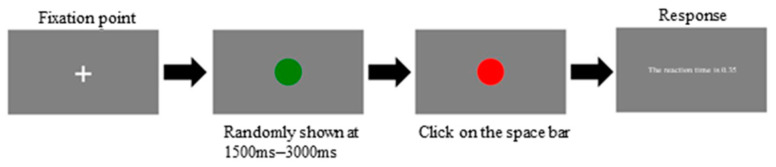
Procedure to obtain the simple reaction time.

**Figure 2:**
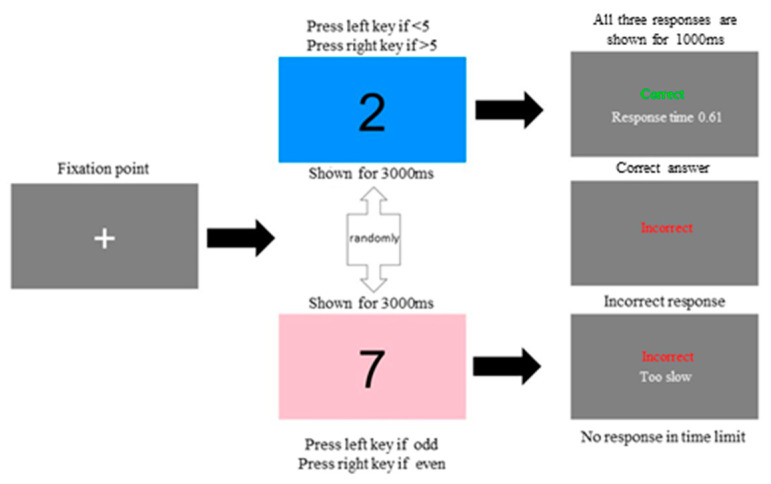
Procedure for choice reaction time.

1. Simple Reaction Time Test (SRT test): Participants were instructed to press the ‘space’ bar immediately when the screen cue changed from green to red. At first, a fixation point (‘+’) was displayed in the center of the monitor for 500 ms. The green cue was shown after the appearance of the fixation point, and it lasted for a random period between 1500 and 3000 ms, whereas the red cue persisted for 3000 ms. Following the response to the red cue, the response time of the previous trial was shown on the screen for 1000 ms (Figure 1).
2. Choice Reaction Time Test (CRT test): Participants were required to identify the background color. The screen randomly showed a blue or pink background with a number in black. In each trial, the fixation point (‘+’) first appeared on the screen for 500 ms. After that, when the screen displayed a blue background, participants were instructed to press the left key if the stimulus number was less than 5; otherwise, they were required to press the right key. When the background color was pink, participants were instructed to press the left key for odd-numbered stimuli and the right key for even-numbered stimuli. The number 5 was not included in either situation. The stimuli appeared randomly, and they lasted on-screen for 3000 ms (Figure 2).

After participants made their decision, a response was shown on-screen. When participants gave a correct response, the word ‘Correct’ was shown on-screen in green. If participants gave an incorrect response, the word ‘Incorrect’ was shown on-screen in red. If participants did not give any decision in 3000 ms, ‘Incorrect’ was shown on-screen in red, and the phrase ‘Too slow’ was shown on-screen in white. Each response to the participants’ answers lasted for 1000 ms.

#### 2.2.2. Dynamic Warm-Up Exercise

The warm-up and protocol procedures were adapted from Eken et al. [55], though several parts were modified, including the high knee pull, straight-leg march, power skip, high glute pull, light high knees, light buttock kicks, a skip, b skip, rapid high knees, carioca, and walking lunge; this culminated in 11 dynamic warm-up exercises in total (Appendix A). Each exercise was performed for 15 s, and a 10 s rest was given between the exercises.

#### 2.2.3. OSE Intervention Group

**Figure A2:**
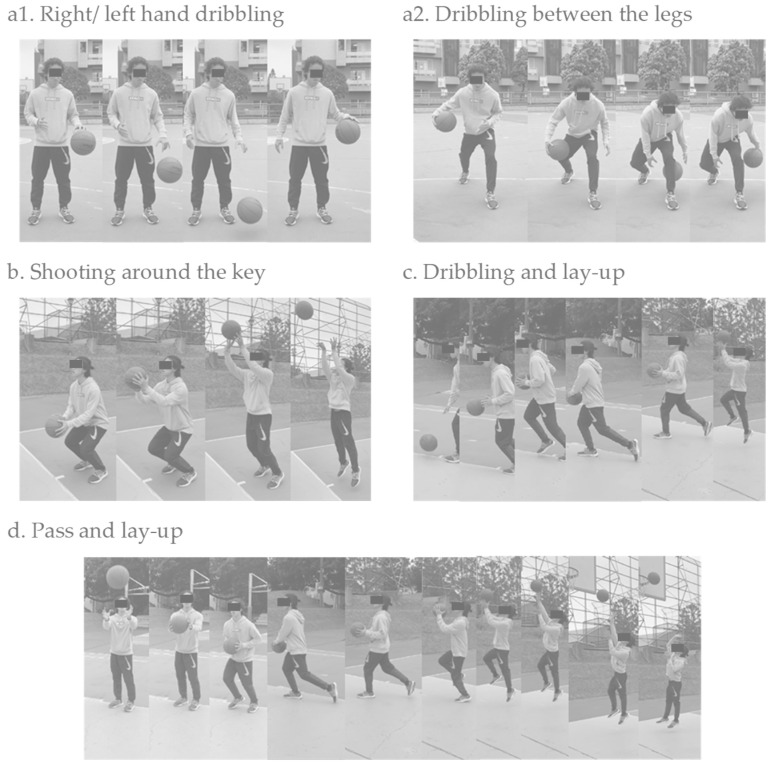
Exercise in basketball exercise intervention.

Participants were guided by researchers to engage in a 30 min basketball intervention session, as outlined in Table 1. (The details of exercise basketball intervention session are shown in Figure A2 and Appendix B). The intervention focused on basketball skills such as dribbling, shooting, and lay-ups. The researchers guided the participants to ensure the correct execution of these movements.

#### 2.2.4. CSE Intervention Group

After the dynamic warm-up, participants were required to run for 30 min on a 400 m running track. The running intensity was determined using the session rating of perceived exertion (RPE), ranging from 1 (rest) to 10 (maximum), in accordance with Foster et al. [56]; this was designed to ascertain the participants’ feelings. In our study, participants were required to maintain an RPE of 4–5–6 for the entire 30 min. To ensure compliance, participants’ RPE levels were confirmed every 5 min, to allow them to better control their exercise intensity.

#### 2.2.5. MSE Intervention Group

A 15 min aerobic running session was conducted, following the same procedures as for the closed-skill exercise after the dynamic warm-up. An RPE of 4–5–6 was also required for each participant so that they could maintain the exercise for the entire 15 min, with the participants checking in every 5 min. Additionally, a 15 min basketball exercise intervention took place after the running session, as outlined in Table 2. All participants followed the exercise intervention procedures, as given by the researchers (Appendix C).

### 2.3. Sampling of Participants

The sampling of participants involved the utilization of online forms, seminars, and posters for recruitment purposes. The sample size was determined using G*Power software (version 3.1.9.4; Düsseldorf, Germany) [57]. The calculation employed an F-test for ANOVA with repeated measures and a between-factor design. To detect a medium effect size (Cohen’s f = 0.25) with a significance level (α) of 0.05 and a power of 80% for observing changes in SRT, CRT, correction rate, and switch cost after intervention [58,59], a total of 120 participants were deemed necessary.

The descriptive statistics of participants are shown in Table 3. Four participants were excluded due to outliers in their declining rate during the CRT test.

### 2.4. Statistical Analysis

The data were analyzed using SPSS 26.0 software (IBM Corp, Armonk, NY, USA). Descriptive statistics are presented with medians, lower and upper quartiles (Q1 and Q3), means, and standard deviations (S.D.). A two-way repeated measure analysis of variance (ANOVA) was conducted to ascertain the simple reaction time, choice reaction time, correction rate, and switch cost (SC). The SC was calculated as the difference between the choice reaction time and simple reaction time. The closed-skill exercise group served as the control group in the analysis. The effect sizes were estimated using partial eta square ($\eta_{p}^{2}$). Effect sizes of 0.04, 0.25, and 0.64 are considered as the recommended minimum effect size (RMPE), moderate effect size, and strong effect size, respectively [60].

If the participants’ declining rates, before and after the CRT test, exhibited an outlier (more than 2 standard deviations), or if the correction rate before or after the CRT test was less than 50%, the corresponding data were excluded; this procedure was adapted from Hughes et al. [61], with some modifications. The significance level was set at α = 0.05. The statistical results were visualized using R (R Core Team, Vienna, Austria) [62] and the ‘ggplot2’ package [63].

## 3. Results

### Differences between Task-Switching Tests

**Figure 3:**
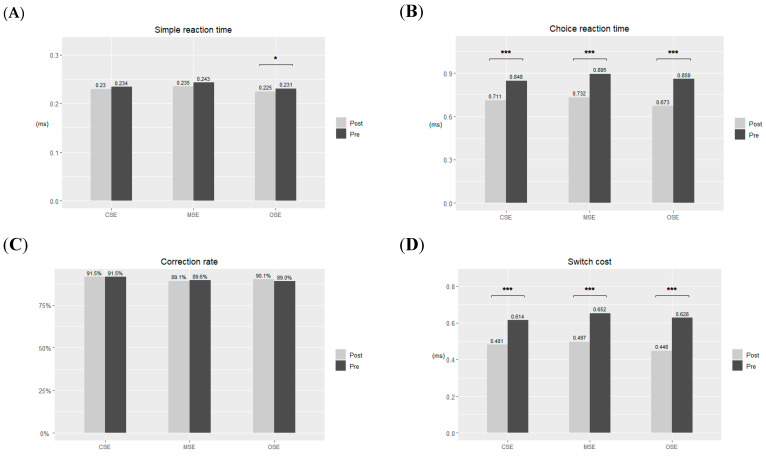
The results of the pre-test and post-test for closed-skill exercise, mixed-skill exercise, and open-skill exercise groups. (**A**) Mean simple reaction time, (**B**) mean choice reaction time, (**C**) mean correction rate in percentage, and (**D**) mean switch cost. * *p* < 0.05, *** *p* < 0.001.

The mean times for the pre- and post-SRT tests are presented in Table 4 and Figure 3. Following analysis using a two-way mixed-design ANOVA, it was observed that only the OSE group demonstrated a significant effect (*F* = 36, *p* = 0.029, Figure 3A) after the exercise intervention took place. No significant effect between groups was obtained but moderate effect size was shown (*F* = 2.079, *p* = 0.130, $\eta_{p}^{2}$ = 0.035).

Regarding CRT, all three groups showed a significant improvement in the post-test (CSE: *F =* 38, *p =* 0.000; OSE: *F =* 36, *p =* 0.000; MSE: *F =* 39, *p =* 0.000; $\eta_{p}^{2}$ = 0.543; Figure 3B). No significant differences were found between the three groups. However, a small effect size was obtained (*F* = 1.528, *p* = 0.226, $\eta_{p}^{2}$ = 0.013).

No significant effects were identified in the correction rate, either between groups (*F* = 1.119, *p =* 0.330) or within groups (*F* = 0.601, *p =* 0.805,$\eta_{p}^{2}$ = 0.001; Figure 3C). A small effect size was found between groups ($\eta_{p}^{2}$ = 0.019).

For SC, the post-test results also significantly improved compared with the pre-test results for the CSE, OSE, and MSE groups (CSE: *F =* 27.656, *p =* 0.000; OSE: *F =* 48.347, *p =* 0.000; MSE: *F =* 47.952, *p =* 0.000; Figure 3D). No significant differences were found between the three groups (*F* = 1.329, *p* = 0.273). Small effect sizes were obtained between groups ($\eta_{p}^{2}$ = 0.009) and in intervention × group ($\eta_{p}^{2}$ = 0.016). A moderate effect size was found within groups ($\eta_{p}^{2}$ = 0.518). The full results of effect sizes are shown in Table 5.

## 4. Discussion

The aim of this study was to investigate the effects of closed-skill exercise (CSE), open-skill exercise (OSE), and mixed-skill exercise (MSE) on executive function. Our results showed that only OSE presented with significant improvements in SRT, CRT, and SC. The other two groups displayed significant enhancements in CRT and SC, but not SRT. Also, most of our subjects showed small to moderate effect sizes. Although no significant differences were demonstrated among the three groups, OSE had the most comprehensive effect on executive function, followed by MSE and CSE.

The results of effect sizes are supported by Verburgh et al. [64] and Park and Etnier [58], showing the relationship between exercise and executive function. Both the between group and the intervention × group values under CRT and SC showed small effect sizes. This further supports our assumption of the linkage between OSE and executive function. However, the opposite result was shown in a previous study [65,66]. Morava et al. [65] found no effect after a single bout of aerobic exercise in task-switching performance under pressure situations. Based on the research of effect size, there is still no conclusion about whether single-bout exercise can effectively acutely improve people’s executive function.

It is evident that after performing the interventions in the three groups, there was a significant difference between the CRT and SC, pre-test and post-test. However, there was no significant difference found between the groups. This aligns with the comprehensive analysis by Zhu et al. [42], but several studies have suggested that OSE may be more effective in enhancing cognitive abilities [29,41,67]. Further research and additional studies are needed to investigate the impact of different types of single-bout exercises on cognitive function, including cross-sectional studies, particularly across various athletes.

Furthermore, significant improvements in SRT were observed, but only in the OSE intervention group, a result that is consistent with the findings of Yu et al. [52]. OSE exhibited significant enhancements compared with SRT, CRT, and SC, indicating that OSE has a superior effect on improving executive function. In line with the study by Takahashi and Grove [68], OSE demonstrated a more pronounced acute effect on inhibitory control as compared with CSE. This might be because OSE induced greater hemodynamics and a more effective activation of the frontal lobe. Additionally, another study suggested that engaging in OSE can enhance inhibitory ability and cognitive flexibility, and it can expedite the updating and transitioning of targets during inhibitory control [42,69]. 

The correction rate showed no improvement after the exercise intervention, aligning with the results of a study conducted by Ellemberg and St-Louis-Deschênes [46]. The correction rate is associated with the dorsolateral prefrontal cortex, which is a part of the frontal lobe [70]. In the task-switching test, participants were required to answer correctly as soon as possible. Therefore, participants did not sacrifice accuracy to achieve a faster response time.

Sport training requires a degree of cognitive function [29]. OSE, such as basketball, adopts different tactics and skills based on varying environments and competitors [67]. This requires high levels of body coordination, motion control, and spatial perception, among other factors. MSE introduces an additional cognitive control component [71]. The task-switching test in our study, which involved perceptual speed, stimulus identification, inhibitory control, and updating working memory, corresponds with the cognitive demands of both OSE and MSE [5,41].

Moreover, Easterbrook’s cue utilization theory suggests that, as an individual’s arousal level increases, their focus becomes more concentrated [72]. Audiffren et al. [73] found that single-bout exercise can elevate arousal levels and accelerate information processing. Additionally, Lennemann et al.’s [74] study indicated that agility training, which places high demands on motor control and coordination, as well as distinct cognitive processes (compared with other exercises), effectively enhances concentration levels.

When testing each aspect of our task-switching test, there were no significant differences observed between the three types of exercise interventions. One plausible explanation for this outcome is the developmental background of the participants. According to Piaget’s theory of cognitive development, children have different cognitive needs at various age stages [75]. Satisfying these cognitive needs at a higher level, at different developmental stages, may influence an individual’s ability to reach a higher cognitive level in adulthood [76]. Moreover, studies on the impact of different types of exercise on cognitive function suggested that cognitive improvements vary depending on the nature of the sport [77,78].

Koch and Krenn also found that elite athletes who engaged in OSE before the age of 18 and transitioned to CSE later in adulthood demonstrated superior cognitive flexibility, working memory, and other parts of cognitive function when compared with elite athletes who continued to engage in OSE in adulthood [77]. Their study also suggested a ceiling effect regarding the cognitive benefits of OSE, indicating that prolonged engagement with such exercises may not continuously stimulate the brain. Therefore, considering the above inferences, the key factor in improving cognitive function may be whether a sport skill introduces new cognitive demands to individuals.

Some recent studies could support our argument. Maurer and Munzert [79] investigated the exercise habits of several basketball and golf players, and they designed a tailor-made experiment comparing familiar internal and external situations with an unfamiliar situation. They found that the athletes’ performance dropped under unfamiliar conditions. This indicates that individuals are unable to perform motor skills automatically when facing unfamiliar situations, necessitating alternative strategies to achieve their targets. Consequently, this process imposes an additional cognitive load on the athlete. The same perspective was reiterated in the review by Tomporowski and Pesce [80], which highlighted the link between motor skill acquisition and cognitive function. The perceived difficulty of the task is a major factor here. Cognitive processes, whether a person is communicating with others or being guided by an instructor, contribute to the cognitive benefits derived from exercise. These studies reinforce the notion that the introduction of new cognitive requirements is the primary element through which exercise enhances cognitive function.

Nevertheless, this study had the following potential limitations:

1. The control group in this study performed CSE, which has been found to improve cognitive function in many previous studies. The reason we chose CSE as our control group is that we wanted a stricter baseline to verify the effect of OSE and MSE, even though this may have impacted the significance of our results. Further research could include a non-exercise group to determine which exercise intervention yielded a greater effect, thereby improving our study design.
2. There were no restrictions on participants who regularly engaged in CSE or OSE over a long period of time. Those past experiences may weaken the effect of our designed intervention. The experimental data may have been influenced by the participants’ exercise habits. However, it is not easy to track participants’ previous exercise habits, classify, and exclude regular exercise participants, which could be improved upon for future research.
3. Some participants may have found our task-switching test easy. In our experiment, some participants found the CRT part difficult, while others perceived it as easy. It was challenging to ascertain the difficulty level of the test for each participant before they completed the entire experiment. Therefore, insufficient complexity may have led to inaccurate assessments of each participant’s initial executive ability and the effects of exercise intervention.

To sum up, the enhanced SRT via OSE may be attributed to the effective activation of the frontal lobe. Furthermore, the improvement observed in CRT and SC, across all three exercise intervention groups, could be linked to heightened arousal levels and accelerated information processing in the brain following the intervention.

## 5. Conclusions and Future Directions

In conclusion, the study findings indicate that all three types of exercises have an acute effect in reducing the choice reaction time and switch cost. Notably, open-skill exercise facilitated a specific reduction in the simple reaction time. Our recommendation is that individuals should incorporate all three types of exercise into their regular routine to promote an acute improvement in executive function. Future research could explore whether learning a new exercise skill may activate the brain or arousal levels more effectively compared with practicing a familiar exercise skill. Additionally, including more non-intervention groups as control groups or conducting a separate analysis by gender may provide further insights.

## Figures and Tables

**Table 1:** Basketball training and time management subjects in the open-skill exercise intervention group.

Training Subject	Training Time	Resting Time
1. Right hand dribbling × 2 times	15 s	15 s
2. Left hand dribbling × 2 times	15 s	15 s
3. Dribbling between the legs × 4 times	15 s	15 s
4. Shoot around the key × 2 time	2 min	3 min
5. Dribbling and lay-up × 3 times	1 min	1 min
6. Pass and lay-up × 5 times	1 min	1 min

**Table 2:** Basketball training and time management subjects in the mixed-skill exercise intervention group.

Training Subject	Training Time	Resting Time
1. Right hand dribbling × 1 time	15 s	15 s
2. Left hand dribbling × 1 time	15 s	15 s
3. Dribbling between the legs × 2 times	15 s	15 s
4. Shoot around the key × 1 time	2 min	3 min
5. Dribbling and lay-up × 3 times	30 s	30 s
6. Pass and lay-up × 5 times	30 s	30 s

**Table 3:** Descriptive statistics of participants.

Group	Closed-Skill Exercise (N = 39)					Open-Skill Exercise (N = 37)					Mixed-Skill Exercise (N = 40)				
	Median	Q1	Q3	Mean	S.D.	Median	Q1	Q3	Mean	S.D.	Median	Q1	Q3	Mean	S.D.
Male/Female	25/14					25/15					24/16				
Age	22	20	23	22	1.82	22	21	23	22	1.78	22	20	23	22	1.97
BMI (kg/m^2^)	21.37	19.83	23.65	21.16	3.06	21.86	20.80	24.57	22.54	3.12	21.34	19.83	23.83	21.51	2.94

**Table 4:** Pre-test and post-test results for the three intervention groups.

Subject	Group	Pre	Post
		Mean ± S.D.	Mean ± S.D.
Simple reaction time (ms)	Closed-skill exercise	0.234 ± 0.025	0.230 ± 0.027
	Open-skill exercise	0.231 ± 0.022	0.225 ± 0.024
	Mixed-skill exercise	0.243 ± 0.031	0.235 ± 0.029
Choice reaction time (ms)	Closed-skill exercise	0.848 ± 0.223	0.711 ± 0.162
	Open-skill exercise	0.859 ± 0.237	0.673 ± 0.131
	Mixed-skill exercise	0.895 ± 0.201	0.732 ± 0.167
Correction rate (%)	Closed-skill exercise	91.5 ± 7.5	91.5 ± 6.5
	Open-skill exercise	89.0 ± 7.6	90.1 ± 7.2
	Mixed-skill exercise	89.6 ± 10.1.	89.1 ± 9.0
Switch cost (ms)	Closed-skill exercise	0.614 ± 0.215	0.481 ± 0.161
	Open-skill exercise	0.628 ± 0.226	0.448 ± 0.124
	Mixed-skill exercise	0.652 ± 0.193	0.497 ± 0.155

**Table 5:** Effect size of different subjects.

Subject	Variables	$\eta_{p}^{2}$
Simple reaction time	Within group	0.076
	Between groups	0.035
	Intervention× Group	0.002
Choice reaction time	Within group	0.543
	Between groups	0.013
	Intervention × Group	0.017
Correction rate	Within group	0.001
	Between groups	0.019
	Intervention × Group	0.006
Switch cost	Within group	0.518
	Between groups	0.009
	Intervention × Group	0.016

**Figure A1:**
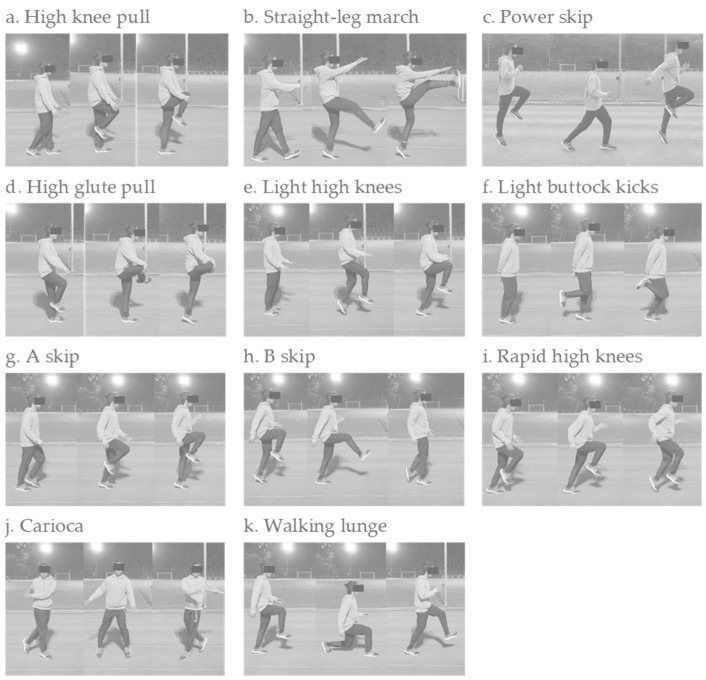
Different exercise in dynamic warm-up.

## Data Availability

Data are contained within the article.

## Appendix A

Dynamic warm-up procedure.

- High knee pull: Use both hands to pull a single knee to the chest while walking. Alternate between legs.
- Straight-leg march: Walk with straight legs, allowing the toes to touch the opposite arm. Alternate between legs.
- Power skip: Jump on one leg while quickly pulling up the other knee. Alternate between legs.
- High glute pull: Pull one ankle up with one hand and the ipsilateral knee up with the other hand, both to stomach height. Alternate between legs.
- Light high knees: Run forward while alternately lifting the knees to waist height.
- Light buttock kicks: Run forward while kicking the heels towards the buttocks, alternating between legs.
- A skip: Take a forward step while lifting one knee, and swing the opposite hand accordingly, alternating between legs.
- B skip: Repeat A-Jumps, and after lifting the knee, extend the calf forward, alternating between legs.
- Rapid high knees: Repeat the light high knees, but increase the overall speed.
- Carioca: Stand with feet in a ‘ready’ position. Start with the body sideways on one end, and the foot closer to the starting point moves laterally towards the endpoint, with the other foot following the lateral movement. After standing upright, the foot closer to the starting point moves laterally towards the endpoint from the backside, with the other foot following the lateral movement. Perform this action facing the left and right sides separately.
- Walking lunge: While walking forward, lift one knee and step down, with the opposite knee touching the ground.

## Appendix B. Basketball Exercise Intervention Instructions

- Dribbling:
  a1: Right/left hand dribbling: Perform simple dribbling using only one hand at a time.
  a2: Dribbling between the legs: Execute dribbling by transferring the ball between one hand and the other, passing it between the legs; also known as a between-the-legs crossover.
- Shooting around the key: Stand and take shots from each designated lane outside the paint area, elbow area, and free-throw line. Complete a total of 11 shots.
- Dribbling and lay-up: Set up two cones, a step away from the elbow area as the starting point. Participants must dribble from one starting point, perform a lay-up, grab the rebound, and dribble to the other starting point. Repeat alternately until the designated time is up.
- Pass and lay-up: A researcher stands in the painted area. Participants pass the ball from the same starting point as in step 3. Subsequently, they run near the basket, catch the ball, and execute a lay-up. After retrieving the rebound, move to the other starting point. Repeat alternately until the designated time is up.

## Appendix C. Exercise Intervention Instructions

- Materials:
  - Computer—Acer a315-55g.
  - Basketball.
  - Cone.
  - Time watch.
- Exercise intervention:
  - Reminder to use the RPE before CSE and MSE intervention.
  - Provide satisfactory rest, depending on the weather and the participants’ condition in the CSE and MSE group.
  - Make sure participants understand how to do the dynamic warm-up and basketball exercise.
- Procedure for CSE:
  - Conduct dynamic warm-up.
  - Reinforce the use of RPE.
  - Initiate 30 min of field running, recording the participants’ RPE every 5 min.
  - After 30 min of field running have been completed, make sure that participants execute the task-switching test within 5 min.
- Procedure for OSE:
  - Conduct a dynamic warm-up.
  - Demonstrate the basketball exercise once before the participants engage in it.
  - After 30 min of the basketball exercise have been completed, make sure participants execute the task-switching test within 5 min.
- Procedure for MSE:
  - Conduct dynamic warm-up.
  - Reinforce the use of RPE.
  - Initiate 15 min of field running, recording the participants’ RPE every 5 min.
  - Guide participants to the basketball court.
  - Demonstrate the basketball exercise once before the participants engage in it.
  - After 15 min of the basketball exercise, ensure that the participants perform the task-switching test within 5 min.
